# Study on Sperm-Cell Detection Using YOLOv5 Architecture with Labaled Dataset

**DOI:** 10.3390/genes14020451

**Published:** 2023-02-09

**Authors:** Michal Dobrovolny, Jakub Benes, Jaroslav Langer, Ondrej Krejcar, Ali Selamat

**Affiliations:** 1 Faculty of Informatics and Management, Center for Basic and Applied Research, University of Hradec Kralove, 500 03 Hradec Kralove, Czech Republic; 2 Malaysia Japan International Institute of Technology (MJIIT), Universiti Teknologi Malaysia Kuala Lumpur, Jalan Sultan Yahya Petra, Kuala Lumpur 54100, Malaysia; 3 School of Computing, Faculty of Engineering, Universiti Teknologi Malaysia (UTM), Skudai 81310, Malaysia

**Keywords:** sperm-cell detection, small-object detection, yolo, computer-aided sperm analysis

## Abstract

Infertility has recently emerged as a severe medical problem. The essential elements in male infertility are sperm morphology, sperm motility, and sperm density. In order to analyze sperm motility, density, and morphology, laboratory experts do a semen analysis. However, it is simple to err when using a subjective interpretation based on laboratory observation. In this work, a computer-aided sperm count estimation approach is suggested to lessen the impact of experts in semen analysis. Object detection techniques concentrating on sperm motility estimate the number of active sperm in the semen. This study provides an overview of other techniques that we can compare. The Visem dataset from the Association for Computing Machinery was used to test the proposed strategy. We created a labelled dataset to prove that our network can detect sperms in images. The best not-super tuned result is mAP 72.15.

## 1. Introduction

One out of every ten couples suffers from infertility [1]. It can have a detrimental impact on a couple’s quality of life and lead to social and psychological issues [2]. Male factor is responsible for over half of all infertility cases [3]. Semen analysis is used to identify male infertility or subfertility and establish treatment options [4]. The shape and size of sperm components are inspected, and the per cent-ages of normal and aberrant sperm are determined in sperm morphology assessment, one of several procedures in semen analysis.

Sperm analysis can be more profound and lead toward DNA analysis [5]. Due to the uncertain efficiency of normal sperm parameters in detecting male factor infertility problems and boosting the success rates of assisted reproductive procedures, additional, comprehensive sperm parameters that could affect male fertility and reproduction have been investigated. Thus, using previously described methods such as single-cell gel electrophoresis (COMET) assay, sperm chromatin structure assay (SCSA), acridine orange test (AOT), terminal deoxynucleotidyl transferase-mediated deoxyuridine (TdT) triphosphate (dUTP) nick end labelling (TUNEL) assay, and sperm chromatin dispersion (SCD), the effects of various However, examining sperm DNA may be difficult due to the unique structure of sperm DNA, which differs from that of somatic cells [6]. Furthermore, during spermatogenesis, sperm DNA undergoes numerous alterations and is compressed by being tightly packaged with various types and numbers of protamines in different species. Despite these challenges, these approaches provide valuable information regarding the causes and consequences of DNA damage in sperm and the consequences of these damages on reproduction.

The vast majority of earlier sperm cell detectors achieved good accuracy since the density was minimal, according to a survey report [7] (only 10–20 sperm cells presents in the video). The accuracy reduces dramatically as the density rises. For example, as described in [8,9], Hamilton Thorne, a commercial computer-based automated system, produces measuring inaccuracies in densely populated sperm suspended due to multiple clashing sperms. Our previous results show possible applications of object detection architectures [10], also as using deep convolutional networks to upscale medical images [11].

In order to address the lack of beef production, the Indonesian government built and mandated artificial insemination centres, such as The Lembang Institute for Artificial Insemination, to provide high-quality frozen bull semen as the primary substance for artificial insemination. As a result, artificial insemination is the most extensively used reproductive technology for increasing beef production in the country [12]. Currently, sperm assessment is done manually at The Lembang Institute for Artificial Insemination.

The head, midpiece, and tail are the three primary sections of a spermatozoon, with the head being separated into acrosome and nucleus [13,14]. Anomalies can occur in any of these areas, although the abnormalities of the head are the most common [15]. The initial stage in automatically detecting head anomalies is to segment the head from the background and into its basic pieces, notably the acrosome and midpiece. The contour information of the sperm head has been proven to be crucial for improving sperm head description, and classification [16]. As a result, precision is crucial while removing the sperm head contour.

Subjectivity, low accuracy, inter variability, and intra-variability are all significant limitations of manual sperm motility measurement [17,18]. Computer-assisted sperm analysis (CASA) has been frequently adopted to circumvent these limitations. However, there are several limitations to employing CASA, especially when evaluating sperm motility in fresh bull sperm, where sperm motility is relatively fast, and partial occlusions are common. Our goal in this study is not to completely replace the current CASA system. We want to improve a precision and multi-sperm cell detection in static images when using computer-assisted methods. The accuracy and speed of multi-sperm tracking are also constrained. The second important issue is the difficulty in accurately classifying motility.

The low accuracy and speed of multi-sperm tracking are major roadblocks. Several researchers have attempted to solve this problem [7]. Sorensen et al. [19], for example, utilized a Particle Filter and a Kalman Filter with a Hungarian algorithm for labelling, which is comparable to the method used by Jati et al. [20]. The authors of Imani et al. [21] used frame difference background subtraction and a non-linear diffusion filter to select the threshold value. The samples in these trials exhibited low sperm densities, with only a few sperm visible in one field of view, and blockage or passing sperm were uncommon.

Previous results in [22] show, as well as employing deep convolutional networks to the geotechnical field. Review [23] demonstrate possible uses of object detection architectures. Furthermore, articles [24,25] describe great use cases of deep neural networks.

This article extends the results and thoughts of [26], where results of YOLOv5 are presented. This study testes architecture of YOLOv5, with its hyper-parameters and configurations on specific tasks. Detection of a sperm cell using new architecture is the main approach of this study.

### 1.1. Topic Overview

**Figure 1:**
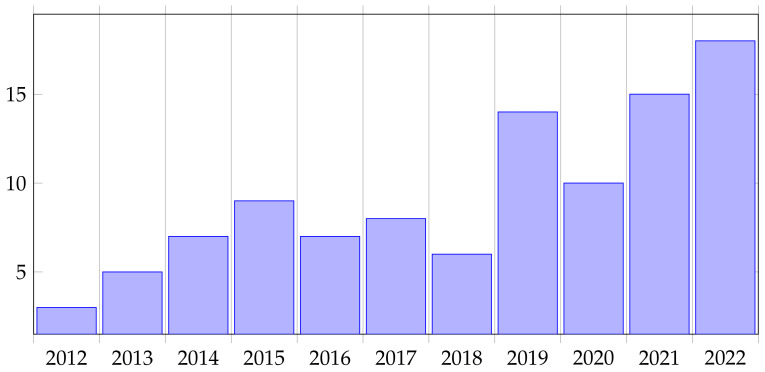
Yearly count of articled published on the Web of Science.

Regarding the Web of Science database, the topic of sperm detection is slightly increasing as shown in Figure 1. We used a query (ALL=(spermdetection)AND(ALL=(computer)ORALL=(vision))NOTALL=(gene)NOTALL=(DNA)). Since 2019 there has been an increase of published articles. Any other criteria did not restrict the search. Results also include conference papers. Indexes included in the search were: SCI-EXPANDED (2995), CPCI-S (272), ESCI (109), BKCI-S (36), SSCI (28), IC (2) and CPCI-SSH (1).

**Figure 2:**
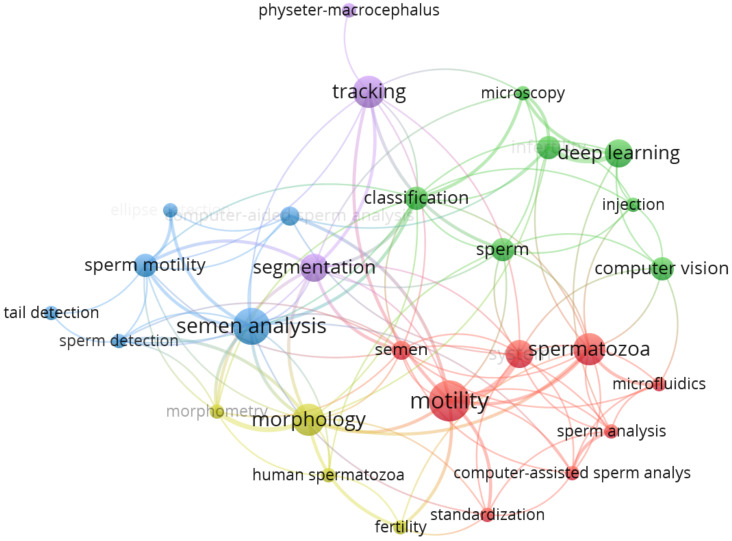
Keyword analysis.

Keyword analysis in Figure 2 shows the connection between sperm detection and other topics; in total, 28 keywords are connected into 5 clusters by 115 links. The amount of all used keywords is 303. The analysis shows the diversity of multiple topics that are around sperm analysis.

### 1.2. Related Works

In [27], the authors focus on segmentation and accurate recognition of the individual parts of the sperm and detailed analysis of the characteristics of the head. The input to the algorithm is an image. The authors used a dataset of twenty images on which they achieved success rates of up to 98% in the process of sperm head recognition.

To segment and identify human sperm cells in an image obtained from a semen sample, the authors of research [28] compare several convolutional neural network (CNN) architectures. In contrast to other investigations, pieces are neither dyed nor washed to enable a complete study of sperm quality, making analysis more difficult owing to clutter. According to their findings, training on entire images is preferable to training on patches when the class skew is appropriately handled. In deep CNNs, full-picture exercise with up-sampling during training improves pixel-wise accuracy and detection performance. The best network gets an accuracy of 93.87%.

In [29], the authors removed unwelcome human variables. Authors develop a hybrid smartphone-based method that completely automates the morphological examination of sperm. The two successive operations in the proposed hybrid system are the automatic segmentation of prospective sperm morphologies and the classification of normal and defective sperms. During segmentation, they were testing grouping into the clusters with and without the sparsity approach and identifying Region of interest (RoI) in the images. They created a dataset based on morphological sperm images which is publicly accessible. The new dataset was used for the classification stage, with labels designating non-sperm, average, and diseased sperm. Using wavelet transform and descriptors, domain-specific features were obtained for the classification stage, where classical and ensemble machine learning methods were then applied. The proposed methods produced classification accuracy results of up to 87%.

The authors of article [30] suggested an algorithm that enables the evaluation of the concentration and sperm motility rate in microscopic movies. Due to the constraints of microscopic images, which include low contrast and noise, the scientists first dealt with pre-processing. Then, a hybrid approach utilizing various segmentation approaches is suggested for accurate sperm detection among noise and debris. The next step was to define sperm concentration using the geometrical characteristics of the sperm head’s bounding ellipse. The inter-frame difference was then used to detect motile sperm. Finally, the proposed method was evaluated using microscopic videos of human sperm, and the outcomes were compared to a manual evaluation performed independently.

In the paper [31], Prabaharan et all. the authors propose CNN for the classification of fertile sperm. The proposed process includes detection, segmentation and morphological analysis. As input, the authors used a public dataset containing 20 images, where each image contains 15 to 20 sperm. The proposed model in MATLAB showed a high success rate, up to 98.99%.

Alabdulla et al. proposed in the paper [32] a new method for sperm cell tracking. The method can handle up to 30 frames per second from different camera sources, depending on the video quality and lighting. The algorithm uses dynamic gates that change size in response to the speed of the sperm cell to determine the shape of each observed object that is used to describe colliding sperms. The technique has a success rate of up to 95% when identifying sperm cells.

Deep sperm is a new technique developed by Hidayatullaha et al. in the publication [33] to identify sperm cells in films. The technique is adapted for recognising small objects and is based on YOLO. Compared to YOLOv3 and YOLOv4, this approach reports superior outcomes. Especially for YOLOv3 by 2% of validation accuracy and YOLOv4 by 0.25%. In YOLOv4, the model displays 95.03 mAP versus 94.12 mAP. The process is up to 10 times faster than R-CNN and produces comparable results. There is no YOLOv5 architecture in this work.

In the paper [34], the authors developed a new computer-aided sperm analysis (CASA) system. The system uses deep learning to automate the identification of sperm in testicular biopsy samples. A deep object detection network, composed of a feature extraction network and an object detection network was trained. The authors used testicular biopsy samples of 30 patients in the form of 702 de-identified images. Preprocessing such as Diffraction correction was also performed on the images. Developed deep learning CASA system achieved a mean average precision (mAP) of 0.741 with an average recall (AR) of 0.376 on their dataset. The system works in real-time.

Authors of study [35] present a method for categorizing sperm based on an examination of their entire anatomy, including the flagella, which they claim are not well visible and are not taken into account by conventional CASA systems. The article’s authors outline the evolution of the Mask R-CNN architecture. They used one of their databases and two that were made available to the public for the study. The resulting technique yields an accuracy of 94.28% mAP for head detection and 90.29% mAP for the complete flagellum. However, Flagella segmentation yielded much worse results (50.88%) than head segmentation (88.32%).

The authors of article [36] developed a CNN model for classifying human sperm heads. More particular, the 16-layer visual geometry group (VGG16) was employed. The dataset consists of 1200 images of human sperm heads classified as healthy or sick. The preprocessing is further described in the paper. Otsu’s thresholding procedure, Area opening method, Image complement process, and Data augmentation are among the algorithms used. As a result, the model obtains a sensitivity of 98.82% and an accuracy of 97.92%.

In [37], Ciara O’Meara et al. deal with the issue of livestock reproduction in connection with sperm quality. Based on a microfluidic chip they developed a portable microscopic imaging system. The system detects sperm vitality and survival rate and based on these values evaluates the sperm quality. Pig sperm samples and eosin-aniline black were used to develop the system. Specifically, sperm samples from 10 boars were collected and mixed. A developed microfluidic chip is responsible for mixing the samples. There was developed an optical system with up to 400 times zoom. This method was used for sperm observation. For identifying sperm motility and survival rate were proposed algorithms based on OpenCV. The system’s pigs’ sperm detection accuracy is 94.0%. The training error was 0.6%. The authors state that the survival rate detection of CASA is 97%. Sperm motility evaluations are consistent with those of computer-aided sperm analysis. A comparison of detection times is also included. For CASA it is 5s. For the method proposed in the paper, it is 9 s.

In the paper [38], the authors introduce a sperm morphology analysis technique named Genetic Neural Architecture Search (GeNAS). The goal of the novel architecture is to detect sperm abnormality. A neural network was created using information from 235 patients who struggled with infertility. There were 1540 sperm images in the dataset termed MHSMA. The authors of the article claim that the majority of methods in use today are built to perform well with carefully picked test datasets. Their GeNAS algorithm is designed to work with general datasets based on real data, particularly class imbalance or low data problems. Accuracy of 91.66%, 77.33%, and 77.66% in the vacuole, head, and acrosome abnormality detection was achieved in the performed experiments.

The authors of article [39] developed the CNN model for classifying human sperm heads. More particular, the 16-layer visual geometry group (VGG16) was employed. The dataset consists of 1200 images of human sperm heads classified as healthy or sick. The preprocessing is further described in the paper. Otsu’s thresholding procedure, Area opening method, Image complement process, and Data augmentation are among the algorithms used. The new neural network model achieves a sensitivity of 98.82% and an accuracy of 97.92%. Comparing those models was also done based on memory costs, training time, FPS, and detection performance. The authors thoroughly outline the scenarios in which the model fails at the end of the article. In their upcoming work, they will continue to incorporate appropriate optimization strategies to improve the functionality of TOD-CNN.

The authors of the publication [40] developed an automated system that evaluates sperm motility parameters. The system was created to speed up the process and get results with greater accuracy and cheaper cost. Fourteen video recordings of bull, ram and stallion semen samples as well as synthetic data were used as a dataset. The process is divided into three main stages. In the sperm detection stage, the main goal is to detect sperm which can be very challenging due to a large number of colliding targets. The authors describe methods such as Optical flow, frame difference, background subtraction, Gaussian filter or Otsu’s thresholding. The authors mention that they used a local adaptive thresholding method to detect static sperm. Since they are stationary sperms, tracking them throughout the next tracking phase is no longer necessary. The second stage is multi-sperm tracking where the objective is to predict the state of one or more objects as time progresses and to determine the precise trajectory of these objects based on their movement patterns. The authors claim that one of the best algorithms for this problem is the joint probabilistic data association filter (JPDAF). It is relatively fast and has a good success rate, but has been shown to be unsuitable for objects such as sperm because of their speed and rapid changes of movement. The authors further describe that instead of using a single motion model, it may be more efficient to use interacting multiple models (IMM). Based on this consideration they proposed an algorithm called IMM-JPDAF. It is a hybrid model for the simultaneous tracking of multiple sperms and is described in detail by the authors in the article. The authors prepared eight different scenarios and compare the results of the global nearest neighbour (GNN), probabilistic data association filter (PDAF), JPDAF, and also three variants of the hybrid model with GNN, PDAF, and JPDAF algorithms. For comparison, the average optimal sub-pattern assignment (OSPA) distance between the actual and estimated paths per 100 Monte Carlo iterations is calculated. Average ranking is also calculated for every model. In every simulated situation, the hybrid algorithm outperformed the other algorithms when paired with JPDAF and GNN.

In the paper [41] Leonardo F. Urbano et al. proposed a fully automated multi-sperm tracking algorithm for measuring motility parameters over time. The algorithm is capable to detect and track simultaneously hundreds of sperm cells in recorded videos. The algorithm uses JPDAF which uses independent Kalman filters to estimate each tracked sperm’s location and speed. Video in MP4 or AVI format with resolution 640 × 480 pixels at 15 fps is used as input to the algorithm. The authors apply preprocessing methods to these images, which are described in detail in the paper. They include a Gaussian filter, Laplacian of Gaussian filter, or Otsu’s method. The accuracy of sperm cell detection is approximately 95%, and the false detection rate is less than 1%. The authors compared the results with five random videos in which they manually detected sperm. The authors also describe in detail how they used and adapted the JPDA algorithm. They have prepared four simulated scenarios to compare the nearest neighbour (NN), GNN, PDA and JPDA algorithms. The authors claim that the JPDA algorithm performs best in the prepared scenarios and that it is suitable for sperm tracking.

## 2. Materials and Methods

### 2.1. Dataset

Multimedia datasets including more than just images or text are uncommon. Open multimedia datasets in medicine are even rarer. Clinical datasets frequently consist only of images or videos. Visem is a dataset that is unique in two aspects. It is a multi-modal dataset that includes movies, biological analysis data, and participant information, for starters. It is made up of anonymised data from 85 distinct people. This dataset is publicly available from https://datasets.simula.no/visem/ (accessed on 18 March 2022) [42].

VISEM comprises information from 85 male participants who are at least 18 years old. Parameters from a routine semen analysis, a video of live spermatozoa, sperm fatty acid profile, the fatty acid composition of serum phospholipids, demographic data, and WHO analysis data are all available for each participant. Due to drift in the initial sample collected. This makes assessing motility challenging for laboratory employees. In addition, due to concerns about the size of the dataset, we opted only to include one video per participant. The video files in the dataset are over 35 terabytes, with each movie lasting between two and seven minutes.

The videos have a 640×480 pixels resolution and a frame rate of 50 frames per second. The dataset includes six CSV files (five for data and one for video-to-participant ID mapping), a description file, and a video folder. Each video file is labelled with an ID, the date of the video capture, and a brief optional explanation. The code of the person who assessed the video using the WHO standard is then included at the end of the filename. VISEM also includes five CSV files for each of the other data sets, a CSV file holding the IDs associated with each video and a text file containing definitions of some of the CSV columns.

Our use-case study needed more exact data about spermatozoa. Using the object detection method requires object position data not provided in the dataset. We decided to mark data on our own. We were using an annotation tool that will export boxes as coordinates used later for training.

**Figure 3:**
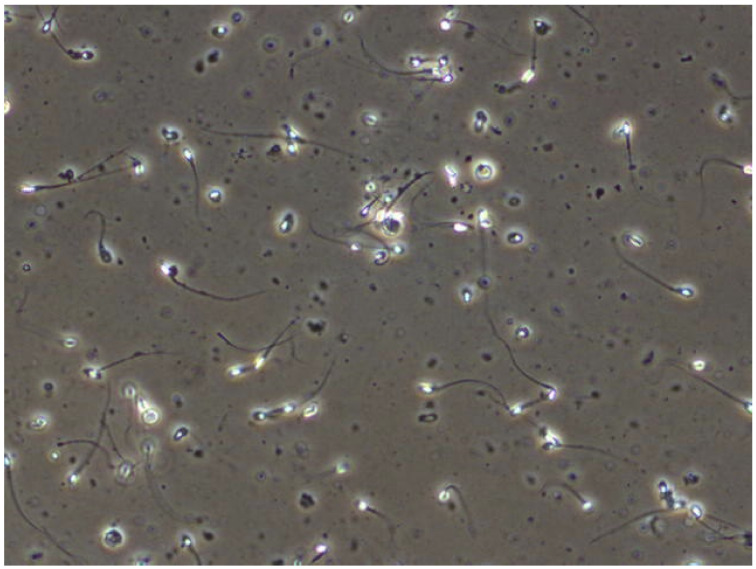
Example image extracted from dataset video [42].

We extracted images from videos in jpg format. The example shown in Figure 3. We reduced the number of images to 382. The distribution of photographs was made similar for each subject.

The annotation tool creates text annotation files with formatting.

(1) 〈object−class〉〈x_center〉〈y_center〉〈width〉〈height〉

where 〈object−class〉 is the object identity, integer number ranging from 0 to (classes−1) and 〈x_center〉〈y_center〉〈width〉〈height〉 is the bounding box specification, float number relative to width and height of image ranging from 0.0 to 1.0.

Since our focus is in this study to determine the possibility to use YOLOv5 architecture, we did not classify sperm cells with any defects. There can be a lot of biological defects. There are defects in heads, midpieces and tails. These defect cells we completely ignore. Sperm morphology detection can be built on top of our current results.

Images also include many artefacts that make this detection hard for deep networks. For example, there can be a blurry image, lousy lighting or wrong contrast.

We split marked images into two datasets. One is for training with a size of 368 images. Second, for validation, that contains 14 images to evaluate the training process.

**Figure 4:**
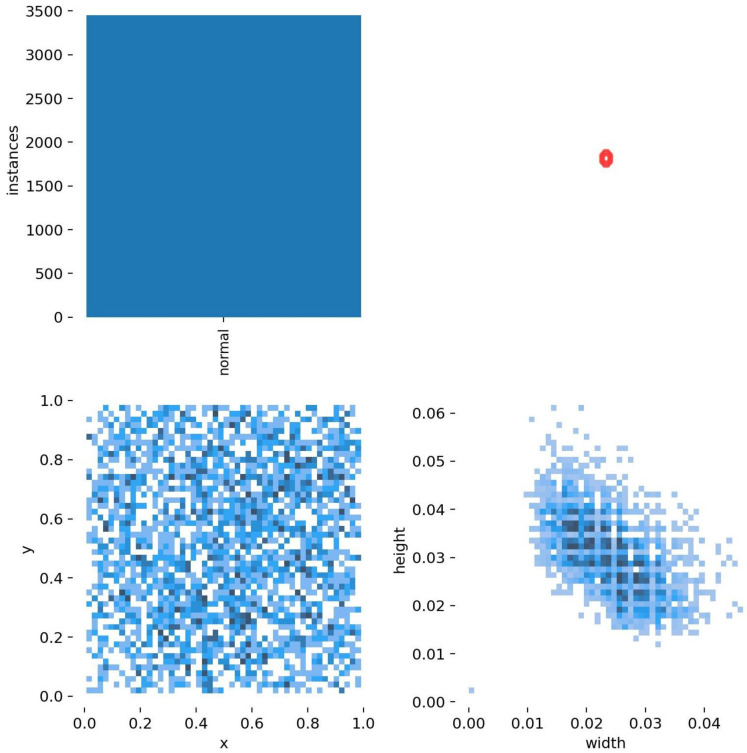
Explained labels and their sizes as width and height of boxes. Plotted with seaborn package.

The dataset we created for training had 3500 labels. The width and height graph is shown in Figure 4.

Regarding the current size of marked images, we will extend the size of both datasets. This approach will lead to better accuracy, lower overfitting, and better performance.

### 2.2. Neural Network Architecture

**Figure 5:**
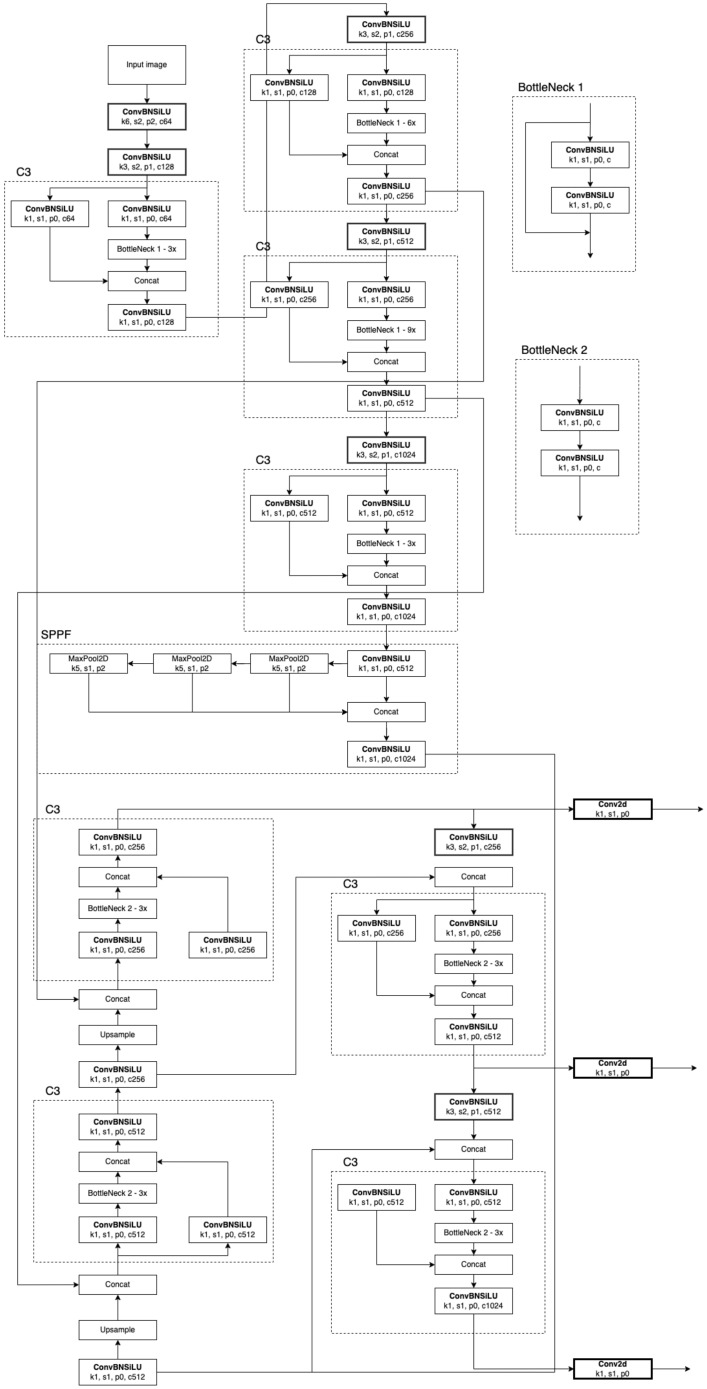
The network architecture of YOLOv5.

YOLOv5 was chosen as our initial learner for three reasons. To begin, YOLOv5 combined the cross-stage partial network (CSPNet) [43] into Darknet, resulting in the creation of CSPDarknet as the network’s backbone [44]. CSPNet solves the problem of recurrent gradient information in large-scale backbones by including gradient changes in the feature map, reducing model parameters and FLOPS (floating-point operations per second), and ensuring inference speed and accuracy while simultaneously reducing model size. In detecting a sperm cell, speed and accuracy are critical, and the size of the model impacts its inference efficiency on resource-limited edge devices. Second, to improve information flow, the YOLOv5 used a path aggregation network (PANet) [45] as its neck. PANet uses a new feature pyramid network (FPN) topology with an improved bottom-up approach to improving low-level feature propagation. Simultaneously, adaptive feature pooling, which connects the feature grid to all feature levels, ensures that meaningful information from each feature level reaches the next subnetwork. In addition, PANet improves precise localization signals in lower layers, significantly improving the object’s location accuracy. Finally, YOLOv5’s head, the YOLO layer, generates three various sizes of feature maps to provide multi-scale prediction, allowing the model to handle tiny, medium, and large objects (Table 1 and Figure 5).

During training, all hyperparameters were set to the same values. Learning rate: 0.01; momentum: 0.937; weight decay: 0.0005; batch size: 8. Pretrained weights were loaded from COCO [46] dataset training.

### 2.3. Hardware

In general, performance requirements for deep learning are very high. On our machine, we have two cards with 7934 CUDA cores. This card is one of NVIDIA’s best-performing cards. We chose NVIDIA cards solely because of the framework support. The graphics clock rate on one of our 1080TI cards is 11,176 MB, with a clock rate of 1607 MHz. Another is a 2080TI, which has 11,019 MB of graphics memory and a maximum clock rate of 1545. The processor used is an i7-8700 with a 3.20 GHz clock speed. Described in Table 2.

In version 3.8.2, we used Python as a programming language. Our Python programming environment was the cli-based script. PyTorch is our main machine-learning framework.

Our environment is built on top of IntelliJ remote development and IntelliJ Idea. We ran the development backend on the server, and the coding was done directly on the machine with remote access.

### 2.4. Validation Methods

Mean Average Precision (mAP) is a metric used to evaluate object detection models. It can have a value between 0 and 1. The formula of mAP is:

$$mAP=\frac{1}{n}\sum_{i=1}^{n}AP_{i}$$

where *i* represents a class, *n* is a number of all classes and $AP_{i}$ is average precision of class *i*.

### 2.5. Results

The best model achieved 72.15 mAP on the validation dataset, comparable to YOLOv4. Table 3 present the comparison of the results. All networks use an input size of image 640 × 480 pixels images.

In a more detailed quantitative investigation (see Table 3). The best-performing model is large. This network achieves a precision of 88.6 per cent, recall of 52.6, and mAP is 72.1. Other networks are achieving lower results. The second best network is nano, with an mAP of 69.6 and precision of 64.7 per cent.

If we compare models by precision only, we get them in order large, medium, nano, small, xtra. So we can determine that in our case, xtra is overfilling.

We can determine that the nano network is too resilient to learn these small objects such as sperm cells. Of course, this also applies to a small network.

#### 2.5.1. Artifact Handling

Artefacts are also a significant source of inaccuracy when it comes to detection. For example, small markings were seen in one of the test samples. They possessed a grayscale similar to that of sperm cells, but they were smaller or defected.

#### 2.5.2. Overfitting Handling

The detectors may overfit if the training dataset’s samples contain too slight a variation. We included samples in the dataset that we believe have enough variety to prevent overfitting in the model. The sperm cells seem relatively little when detected with a magnification of 100×. Therefore having annotated samples is generally limited. To investigate the impact on accuracy and decide which model has the best generalization ability, we add a single dataset split with low variation in the training data.

## 3. Results

This study tested a deep neural network architecture, with its hyper-parameters and configurations detailed in the material and methods section. The detection of a sperm cell is the main target of the study. It was unaffected by partial occlusion, artefacts, many moving objects, the small size of the objects, low contrast, low video resolution, fuzzy objects, and a variety of lighting conditions.

**Figure 6:**
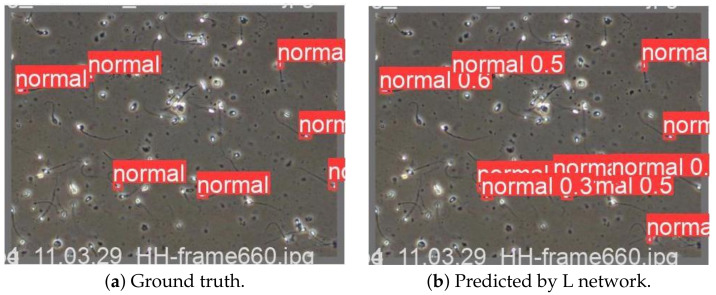
Comparison for validation dataset labels of (**a**,**b**). Generated during training-best epoch.

To summarise, the proposed method performs well in precision, speed, and resource use. On the validation dataset, the mAP was 72.15. However, the tested method uses a significant amount of memory. Furthermore, one of the networks ended up overfitted. The full comparison is available in Table 3. Therefore, we will investigate the training dataset profoundly and try to propose a solution for this xtra network size. Figure 6 presents the comparison of random single image prediction from validation data.

The used dataset is an excellent opportunity to provide data for object detection in terms of sperm detection [42]. In the future, we will also focus on using another dataset to make this work better compared with other methods.

The results can provide great information for future applications such as the automatic determination of infertility.

## 4. Conclusions

Compared to most articles, our article is dedicated to detecting only healthy, non-defective sperm. However, this can be misleading in a context where, for example, the sperm is filmed from the non-defective side. In other words, the position of the sperm can hide defects. The method we used takes only static images. This is because fertility also affects sperm motility. A static image cannot capture this fact. However, mobility is also, in most cases, related to tail defects, which can already be found in a static image. The detection of sperm in human semen is not as well-researched a topic as, for example, the detection of animal sperm.

During the implementation of our experiments, the new YOLOv7 architecture was introduced. This architecture shows very interesting results in the field of object detection. The most interesting change in our view is the improvement in accuracy. Therefore, this architecture will be included in our future work.

As the following steps, we would like to extend the detection to specific defects in sperm heads and tails. This area offers exciting problems to solve.

## Figures and Tables

**Table 1:** Described models in numbers of size.

Model	Nano	Small	Medium	Large	Xtra
Input size	640 × 480				
Number of layers	270	270	369	468	567
Number of parameters	1,765,270	7,022,326	20,871,318	46,138,294	86,217,814
Memory size	0.93 GB	1.73 GB	3.2 GB	4.97 GB	7.34 GB

**Table 2:** Hardware specification of training machine.

Processor	Intel (R) Core™ i7-8700 3.20 GHz (6xCORE)
RAM	16 GB × 4 (2666 MHz) CL13
GPU	GeForce GTX 1080TI (11,176 MB) 1607 MHz
GPU	GeForce RTX 2080TI (11,019 MB) 1545 MHz

**Table 3:** Results achieved on validation dataset in percents.

Model	Nano	Small	Medium	Large	Xtra
Precision	64.7	61.6	71.7	88.6	64.6
Recall	61.4	64.9	57.8	52.6	71.9
mAP	69.6	64.6	66.4	72.1	68.6

## Data Availability

Data can be shared upon request to the corresponding author.

## Abbreviations

The following abbreviations are used in this manuscript:

AOT	Acridine orange test
AR	Average recall
CASA	Computer-assisted sperm analysis
CNN	Convolutional neural network
COMET	Single-cell gel electrophoresis
CSPNet	Cross-stage partial network
CUDA	Compute Unified Device Architecture
DL	Deep learning
dUTP	Deoxyuridine triphosphate
FPN	Feature pyramid network
GeNAS	Genetic neural architecture Search
GNN	Global nearest neighbor
IMM	Interacting multiple models
JPDAF	Joint probabilistic data association filter
mAP	Mean average precision
ML	Machine learning
NN	Nearest neighbor
OSPA	Optimal sub-pattern assignment
PANet	Path aggregation network
PDAF	Probabilistic data association filter
RoI	Region of interest
SCD	Sperm chromatin dispersion
SCSA	Sperm chromatin structure assay
TdT	Terminal deoxynucleotidyl transferase
TUNEL	Terminal deoxynucleotidyl transferase-mediated dUTP nick end labeling
VGG16	16-Layer visual geometry group
